# Improved cotton yield: Can we achieve this goal by regulating the coordination of source and sink?

**DOI:** 10.3389/fpls.2023.1136636

**Published:** 2023-03-29

**Authors:** Aizhi Qin, Oluwaseun Olayemi Aluko, Zhixin Liu, Jincheng Yang, Mengke Hu, Liping Guan, Xuwu Sun

**Affiliations:** State Key Laboratory of Crop Stress Adaptation and Improvement, State Key Laboratory of Cotton Biology, Key Laboratory of Plant Stress Biology, School of Life Sciences, Henan University, Kaifeng, China

**Keywords:** cotton, crop yield, coordination, regulation mechanism, source–sink relationship

## Abstract

Cotton is one of the major cash crops globally. It is characterized by determinate growth and multiple fruiting, which makes the source–sink contradiction more obvious. Coordination between source and sink is crucial for normal growth, yield, and quality of cotton. Numerous studies reported how the assimilate transport and distribution under varying environmental cues affected crop yields. However, less is known about the functional mechanism underlying the assimilate transport between source and sink, and how their distribution impacts cotton growth. Here, we provided an overview of the assimilate transport and distribution mechanisms , and discussed the regulatory mechanisms involved in source-sink balance in relation to cotton yield. Therefore, this review enriched our knowledge of the regulatory mechanism involved in source–sink relationship for improved cotton yield.

## 1 Introduction

Mason and Maskell (1928) were the first to propose the concept of the source–sink relationship, and since then, it has been one of the trending research areas in crop physiology, as it helps target maximum yield (Mason and Maskell, 1928). The source acts as a transient storage organ, where assimilates are exported to the sink for growth. To this end, the sink can be considered as the consumer and importer of the synthesized assimilates (White et al., 2016). In cotton, the mature leaf acts as the source organ that synthesizes the assimilates, which then are transported to the sink organ (flowers, boll, and fibers) *via* vascular tissues (Mangi et al., 2021; Yadav et al., 2022). Source-to-sink photosynthate transport involves the loading of assimilates at the source, followed by its transport and unloading at the repository end (sink), where the sucrose is utilized for plant growth. The main transportation organ is the vascular system, which transports water, minerals, and organic nutrients (Han, 2015).

Carbon (C) and nitrogen (N) in the form of sucrose and amino acids are essential for plant growth and development. Since alterations in the source-to-sink organs greatly affect plant growth, a balanced distribution of the assimilates between them is crucial for attaining maximal crop yield (Zhang et al., 2022). He et al. (2005) found that the storage capacity of maize (*Zea mays* L.) was positively correlated with the total vascular bundle area. Nie et al. (2020) affirmed that a coordinated transport and distribution of the source–sink assimilates is crucial for boll development and improved cotton yield.

Cotton (*Gossypium* spp.) is an important source of natural fiber. Therefore, cultivating high-yielding cotton cultivars has always been the focus of both breeders and researchers (Zhang et al., 2016a). Cotton is mainly characterized by its indeterminate growth system and multiple fruiting. This growth pattern could alter the allocation of the assimilate between the vegetative and reproductive plant parts (Ul-Allah et al., 2021). The imbalanced assimilate distribution subsequently limits the fiber growth and yield (Zhang et al., 2022) and is mostly triggered by diverse environmental cues. This increased the sucrose accumulation in the source leaves and disrupt their transport to the sink (Du et al., 2020; Mangi et al., 2021), thereby limiting growth and yield. Most studies have mainly focused on how the assimilate transport and distribution under varying environmental cues are affecting crop yields (Pilkington et al., 2015; Cui et al., 2019; Bhattacharya and Kundu, 2020; Du et al., 2020). Unfortunately, knowledge about the regulatory mechanism of the source–sink relationship is quite limited (Sonnewald and Fernie, 2018). Although numerous studies have focused on the key regulators of the assimilate transport and distribution in different crops (Bezrutczyk et al., 2018; Lu et al., 2020), little is known about the functional mechanism underlying the assimilate transport between the source and sink, and how their distribution impacts cotton growth. Therefore, we discussed the carbon/nitrogen assimilate transport and distribution mechanisms, the functional mechanisms involved in the source–sink relationship, and their relation to cotton yield.

## 2 Improving photosynthesis is fundamental to cotton productivity

Photosynthesis forms the basis for photoassimilate production; thus, increasing plant photosynthetic efficiency becomes a prerequisite for improving cotton yield (Yao et al., 2017; Yang et al., 2019; Faralli and Lawson, 2020). At the genetic level, previous studies have established a link between photosynthesis and cotton yield; however, fewer recent findings have explored the contribution of natural genetic variation in leaf photosynthesis to cotton productivity. Pettigrew and Gerik (2007) launched a comprehensive review on the prospect of improving photosynthesis using two different approaches. The first is the genetic diversity and the other is the management practices that affect cotton growth or eliminate the negative impact of environmental cues. Studies have addressed the genotypic differences in leaf gas exchange (CER) (Elmore et al., 1967; Pettigrew and Turley, 1998; Clement et al., 2013), specific leaf weight (SLW) (Lei et al., 2022), and ribulose-1,5-bisphosphate carboxylase oxygenase (Rubisco) activity (Benedict et al., 1981) among *Gossypium* species. However, owing to the complexity in the regulation of photosynthesis, attempts to use genetically improved photosynthesis as a bait for cotton yield increase has been challenging. Improving one of these photosynthetic components may prove futile if the rate of another component remains limited throughout the overall process (Pettigrew and Turley, 1998). Thus, cotton breeders should consider the genetic diversity of these photosynthetic components to successfully improve both photosynthesis and yield. With that, the combination of all these components can be configured to improve photosynthesis, which synthesizes assimilates to be partitioned for sink growth.

Although cotton photosynthesis has been correlated with yield at the genetic level, such relationships can only be actualized by favorable climatic or environmental factor during growth (Pettigrew and Turley, 1998). A good example is the reduction in CER in the afternoon, ascribed to photoinhibition, and carbohydrate feedback inhibition. Cornish et al. (1991) reported that the genotypic difference in cotton leaf CER observed in the morning faded away in the afternoon. Genotypes that are able to sustain their photosynthesis at noon would have more photosynthate reserves for cotton growth and development than other genotypes that inefficiently utilize the afternoon sun. Yang et al. (2019) compared the photosynthetic features of early-maturing cotton cultivars from different breeding eras. The crew found that era 3 and 4 cultivars have greater canopy apparent photosynthetic (CAP) rates during the mid- and late reproductive stages compared with the earlier cultivar (era 1 and 2), and such improvement contributed to yield an increase in era 3 and 4 cultivars. Taken together, genotypic diversity in these components such as CER, CAP, and SLW, which often depends on the environmental or growth conditions, makes up the photosynthetic system. Enhancing photosynthetic efficiency directly improves assimilate source supply to feed the growing sink demand.

## 3 Photosynthesis and cotton yield response to elevated CO_2_: Free-air CO_2_ enrichment as an experimental tool

The surge in carbon dioxide (CO_2_) concentration is one of the trending atmospheric changes in the past five decades (Prentice et al., 2001). In the recent years, researchers have become increasingly interested in how plants respond to rising CO_2_ under natural and controlled environments (Allen et al., 2020; Baker et al., 2022). Generally, changes in CO_2_ affect cotton growth and productivity. A rise in CO_2_ enhances photosynthesis, numbers of leaves, and total dry biomass (Ainsworth and Long, 2005; Mollaee et al., 2019). Reductions in stomatal conductance and the shedding of bolls increase CO_2_ on cotton and improve cotton productivity in the process (Mollaee et al., 2019; Li et al., 2021). Most of the conclusions drawn from these studies are targeted at plants exposed to controlled or enclosed environments (Ainsworth and Long, 2005). While the results of these studies form the basis for our current understanding on physiological responses to increased CO_2_, there still exist limits to employing enclosure systems for examining the effects of elevated CO_2_ on plants (Ainsworth and Long, 2005). An enclosed system may produce chamber effects and also trigger the repression of photosynthesis and yield (Morgan et al., 2001), making it not totally suitable. However, among other experimental methods of predicting crop response to elevated CO_2_, the free-air CO_2_ enrichment (FACE) tool has been considered as “the accepted standard” since FACE experiments are conducted in a field environment (Allen et al., 2020; Baker et al., 2022). Unlike the chamber method, FACE eliminates the potential chamber effects (Kimball et al., 1997). In a meta-analysis study, Ainsworth and Long (2005) collected and averaged all the plant physiological and yield responses across all 12 FACE experiments. They found that plants exposed to elevated CO_2_ had a 31% yield increase in the light-saturated leaf photosynthetic rate and a 28% increase in diurnal carbon assimilation. In addition, cotton seed yield increased by almost 40% under elevated CO_2_ conditions (Naikwade, 2020). Until recently, the FACE experimental tool has been widely accepted such that no empirical study has been done to compare FACE with the on-site chamber system or a non-FACE system in cotton (Allen et al., 2020). Allen et al. (2020) compared the outcome of photosynthesis and cotton seed biomass in CO_2_-enriched plants exposed to FACE and non-FACE systems. CO_2_ fluctuates in FACE; thus, photosynthesis and growth decreased under fluctuating CO_2_, as such plant response in FACE is likely to overshadow the benefits of elevated CO_2_ (Allen et al., 2020). Furthermore, the crew found that plant response to elevated CO_2_ in the FACE experiment was lower than that in the chamber experiment. Since results from FACE studies were obtained under natural or field conditions and not in a controlled system, the future prospect of cotton productivity might be limited under such conditions. Taken together, a better understanding of strategies in improving photosynthesis could enhance assimilate synthesis at the source leaves, as these synthesized assimilates are needed to feed the growing sink demand.

## 4 Effects of source–sink regulation on growth and development in cotton

The source–sink relationship is intricately intertwined, and its manipulation affects the sink’s size and strength (Mangi et al., 2021). In cotton, the subtending leaf (mostly considered as the source leaf) houses the carbon (energy source), which is subsequently remobilized (sucrose form) to the boll (sink organ) for fiber development since the sink mainly fixes the remobilized assimilates from the source tissues for their growth and development (Liu et al., 2015). Accordingly, increasing the source (subtending leaf) capacity is one promising way to meet the growing sink demand. Reports have shown that reductions in the source capacity have decreased photosynthesis, inhibited the assimilate transport to the sink, and ultimately reduced the fiber (sink) yield (Cui et al., 2019; Ding et al., 2019). However, whether sink regulation has a significant feedback effect on the source activity remains unknown.

Cotton is characterized by the indeterminate growth type, and is highly susceptible to drought stress during its reproductive phase (Pilon et al., 2019; Zhao et al., 2019). Hence, the partitioning of assimilates between the vegetative and reproductive branches is greatly affected by such conditions. Although plants usually have an adaptive strategy to balance the source-to-sink regulation, drought stress in cotton alters such equilibrium by affecting the source capacity and disrupting the assimilate transport to the fruiting branches (FBs), where it is required for growth. Additionally, drought stress represses sucrose and starch accumulation by reducing sugar-related enzymatic activities (Loka et al., 2020). Consequently, photosynthetic reductions and fiber retardation become more evident in the upper FBs than in the lower branches and, finally, in both FBs (older and the newly formed bolls) (Wang et al., 2016; Zhao et al., 2019). However, Shareef et al. (2018) argued that assimilates are first partitioned to the cotton root, followed by the vegetative and reproductive parts, under drought stress. Furthermore, within the bolls, more assimilates are allocated to the seed than to the fiber, thus reducing fiber development (Tang et al., 2017; Gao et al., 2020; Ul-Allah et al., 2021). Regarding the relationship between subtending leaves (source) and the flower (sink) under drought stress, the subtending leaf accumulated more proline than carbohydrates (Pilon et al., 2019), which reduced the dry matter accumulation and limited fiber development (Pilon et al., 2019). This indicates that drought affects the source-to-sink relationship by inhibiting the assimilate synthesis and their distribution to the sink. Although the relocation of assimilates to the cotton leaves and roots is the main adaptive survival mechanism, it instead decreases the assimilate accumulation at the sink (fiber, boll, and flower), which significantly reduces fiber development and yield. Therefore, efficient assimilate transport and distribution under favorable conditions becomes imperative for improving the source capacity and sink (fiber) development.

Among other factors, plant topping and vegetative branch (VB) removal are considered crucial to cotton growth and productivity, as both exert great influence on source-to-sink coordination (McGarry et al., 2016; Nie et al., 2021). Plant topping could either be manual or chemical-based topping. Both plant toppings promote cotton photosynthetic capacity and leaf area index (LAI) relative to the non-topping. The enhanced LAI is attributed to the increased carbon assimilation rate conferred by plant toppings (Nie et al., 2021). In addition to photosynthesis, plant topping also strongly regulates source–sink photoassimilate partitioning (Xing et al., 2018; Nie et al., 2020). Unlike non-topping and chemical topping, manual topping significantly diverts more photoassimilates towards the FB-sourced bolls, and less assimilate to the redundant cotton bolls, making manual topping more advantageous than chemical topping (Nie et al., 2021). Although chemical topping requires less labor input compared with manual topping, chemical topping shows no advantage over manual topping in terms of seed cotton yield output (Nie et al., 2021).

Available information regarding the impact of VB retention or removal on cotton productivity is contradictory. It is well known that excessive growth of VB often culminates into a large plant structure, making cotton management and harvesting a difficult task (McGarry et al., 2016). The vigorosity of the VB also leads to competition for light, nutrients, and assimilates between the source (stem) and sink organ (FBs), thereby reducing the lint yield (Zhao et al., 2017). Hence, vegetative branching removal is widely applied to cotton cultivation practices in some parts of the world (Zhang et al., 2019). Contrary to the popular notion of growth arrest conferred by VB retention, some other studies have affirmed that VB retention did not reduce seed cotton yield at lower plant density (Dong et al., 2005; Dai et al., 2014; Dong et al., 2017; Nie et al., 2021). Nie et al. (2021) opined a marked increase in the biological yields of the retained vegetative cotton branches compared with the expunged VB at low plant density. Such yield increase was attributed to the increased carbon assimilation capacity in VB retention-sourced plants. Since VB removal is labor intensive and the yield is comparable with the retained VB at low plant density, VB removal can be nullified provided the plant population density is low. Furthermore, VB retention affects photosynthesis and photoassimilate partitioning between the source and sink, thereby impacting cotton growth and productivity. The retention of VB often leads to increases in leaf area index, root growth, and carbon assimilation rate at later developmental stages of cotton. Compared with the VB removal, plants retaining VB have more of their photoassimilates partitioned to the vegetative organs and redundant FB, with less assimilate partitioned to active bolls (sink organ) at the later developmental stages of cotton. This is an indication that VB retention often increases leaf source capacity at low plant density (Hezhong et al., 2007; Nie et al., 2021). Although VB contributes indirectly to economic and biological yield by setting bolls, the qualities of its bolls are poor compared with those of FB-sourced bolls (Nie et al., 2021).

## 5 Mechanisms of carbon transport and distribution in plants

**Figure 1:**
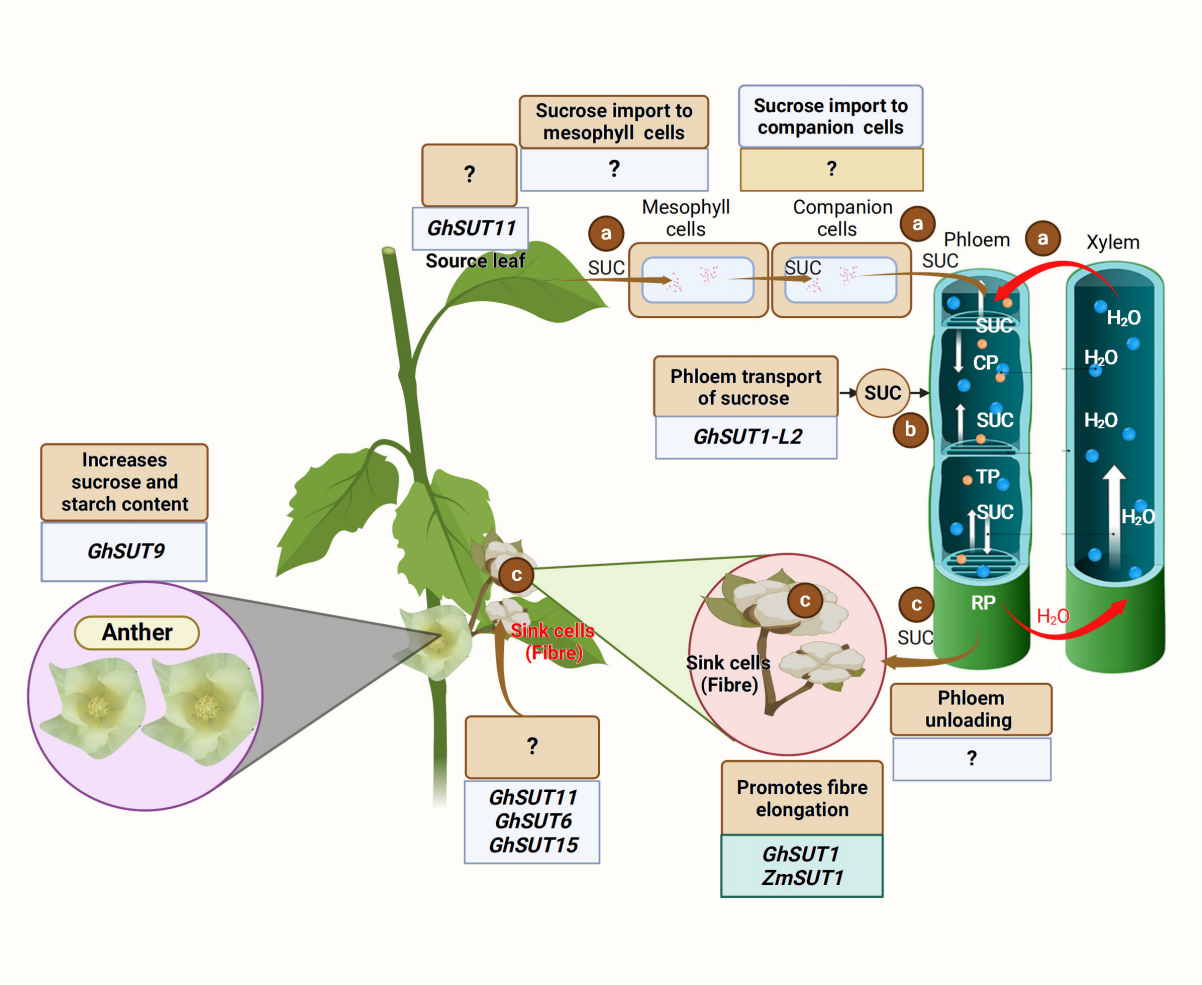
Schematic illustration of the source to sink carbon transport. **(A)** Sucrose is translocated from the mesophyll cell to the companion cells, and finally to the sieve elements of the collection phloem (CP) under a high concentration gradient. **(B)** Sucrose is moved from the collection phloem (CP) to the transport phloem (TP) and finally reaches the release phloem (RP) *via* the activities of sucrose transporters, including SWEET and SUTs. **(C)** The release phloem unloads the sucrose at the sink organ under a low concentration gradient for fiber development. Chao et al. (2020) revealed that *GhSUT11* is expressed in the source and sink organ, but its function is unknown. The crew also found that *GhSUT6* and *GhSUT11* were expressed in the sink organ, but the function of these genes in sucrose transport is unclear (Chao et al., 2020). The genes regulating sucrose transport from leaf to mesophyll cells and from mesophyll cells to the companion cells are yet to be identified. *GhSUT1-L2* is involved in the phloem transport of sucrose from the leaf; however, the gene responsible for phloem unloading is unknown. Recently, Yu et al. (2023) showed that *GhSUT9* expressed in the petal (sink organ) enhances starch and sucrose content in the anther. However, *GhSUT1* and *ZmSUT1* promote cotton fiber elongation (Guo et al., 2017; Ding et al., 2019).

**Figure 2:**
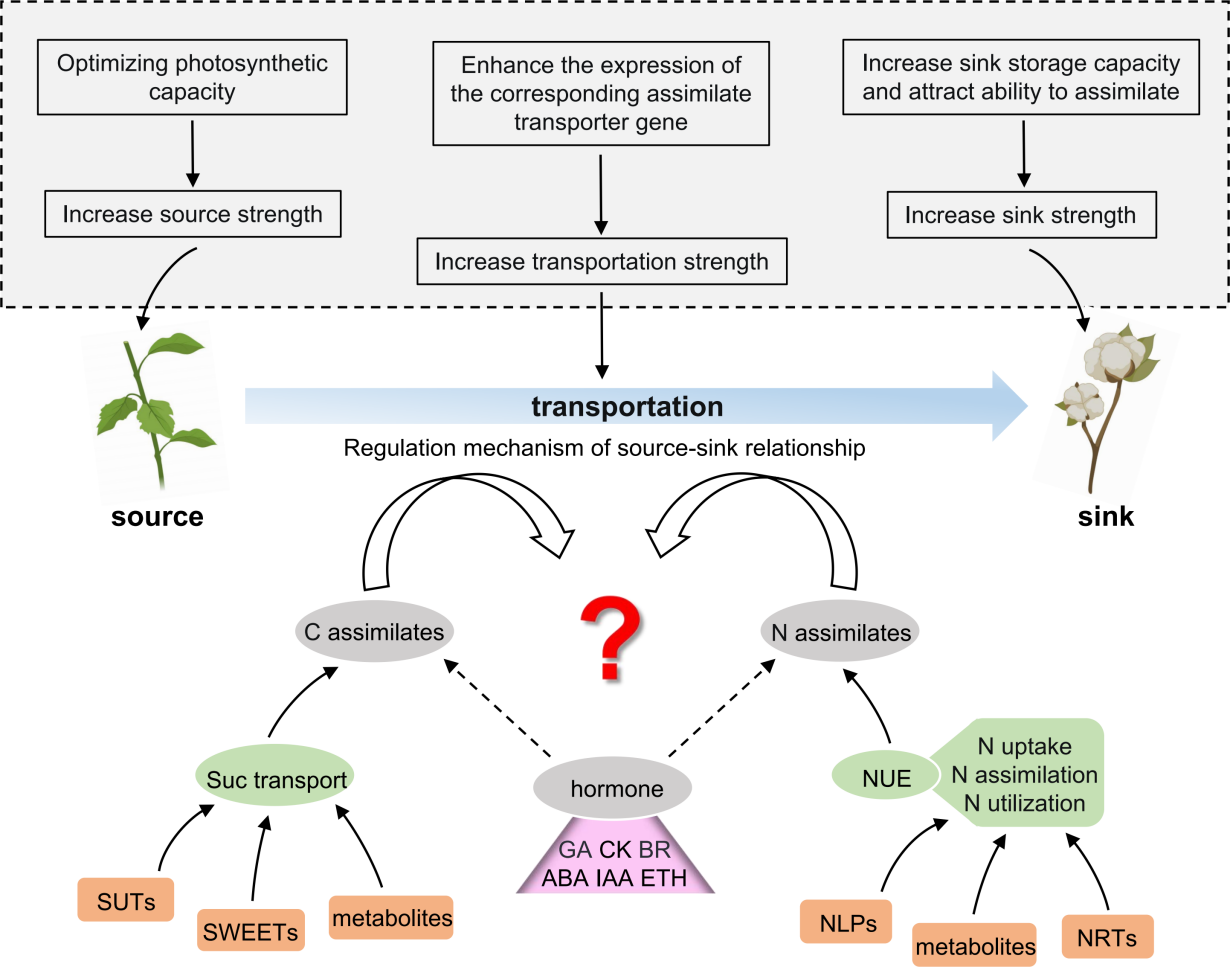
Future prospects of studying the source–sink coordination in cotton. The upper part of the picture (in the dotted box in the gray background) shows several solutions for modulating the source–sink to improve cotton production. The lower part of the picture indicates the main breakthrough steps to predict the regulatory mechanism of the source–sink relationship in cotton, which is as follows: (1) screening and identification of the key genes and metabolites affecting sugar transport and distribution; (2) screening and identification of key genes and metabolites affecting the nitrogen use efficiency (NUE); (3) elucidating the internal mechanism of how plant hormones participate in the regulatory network of the source–sink relationship. C, carbon; N, nitrogen; Suc, sucrose; SUTs, sucrose transporters; SWEETs, SUGAR WILL EVENTUALLY BE EXPORTED TRANSPORTERS; NUE, nitrogen use efficiency; NRTs, nitrate transporters; NLPs, nodule-inception-like proteins; GA, gibberellic acid; CK, cytokinin; BR, brassinosteroid; ABA, abscisic acid; IAA, auxin/indoleacetic acid; ETH, ethylene.

Carbon in sucrose form is transported from the source to the sink organ *via* the phloem (Lemoine et al., 2013). The export of carbon assimilates from the source leaves begins with phloem loading. To date, there are three recognized active phloem loading mechanisms: (1) symplastic loading route, where sucrose leaves the mesophyll cell and moves through the plasmodesmata from one companion cell-sieve element (CC-SE) complex to another *via* SUGAR WILL EVENTUALLY BE EXPORTED TRANSPORTERS (SWEETs) activity (**Figure 1**); (2) apoplastic phloem loading, where high concentrations of sucrose in mesophyll enter the CC-SE complex through a concentration gradient (Zhang and Turgeon, 2018) (**Figure 1**) *via* proton motive force triggered SUCROSE TRANSPORTERS (SUTs); and (3) polymer trapping, where sucrose is converted to larger sugar polymers and unable to diffuse back into the mesophylls. These phloem loading routes utilize the phloem sections (collection, transport, and release phloem) to effectively unload the sucrose. Sucrose is first loaded into the collection phloem, transported through the transport phloem, and finally unloaded *via* the release phloem (**Figure 1**) (Van Bel, 2021; Jin et al., 2022). Sugar transporters, especially the SWEET and SUT proteins, are crucial in the sucrose transport and distribution to the sink (Guo et al., 2021; Ko et al., 2021). SWEET proteins perform bidirectional sugar transport (Chen et al., 2012a). SUTs are sucrose/proton co-transporters that transport sucrose using the energy stored in the transmembrane proton gradient generated by H^+^-ATPases (Gaxiola et al., 2007).

The transport and distribution of photosynthetic assimilates from the source to the sink is essential for proper plant growth, development, and metabolism (Braun et al., 2014). A recent study found GhSUT1-L2-mediated apoplastic phloem loading to be the main mode of phloem loading in cotton (Yadav et al., 2022). Therefore, the rapid expansion of cotton fiber cells requires a continuous supply and sufficient accumulation of sugar.

## 6 Mechanism of nitrogen transport and utilization at the sink level

Cotton yields depend on the N-fertilizer application for its growth and yield (Shah et al., 2017). N is involved in chlorophyll synthesis and enhancing the photosynthetic efficiency of leaves in the bolling stage, thus showing that the coordination between sucrose (transportable carbon form) and amino acids (transportable nitrogen form) is critical for improving the cotton yield (Chen et al., 2012b). The N concentration can affect photosynthesis *via* diverse mechanisms. It is an important component of chlorophyll, the Rubisco enzyme, and is also involved in the development of another photosynthetic organ. Therefore, it is considered an important component of photosynthetic carbon metabolism (Huppe and Turpin, 1994; Stitt et al., 2002). Nitrogen deficiency adversely affects the lint yield by reducing stem elongation (Chen et al., 2020), leaf expansion (Chen et al., 2018), and biomass production (Fritschi et al., 2003). Inorganic N is assimilated into amino acids *via* a series of N-assimilatory enzymes (Tegeder and Masclaux-Daubresse, 2018). After conversion, the root utilizes the synthesized amino acid, while others are remobilized to the source leaves *via* the xylem. Upon arrival, the amino acids are utilized for protein synthesis, albeit lesser amino acid is required for leaf metabolism (Iqbal et al., 2020). Therefore, the amino acid is loaded into the phloem and then remobilized to the sink organ. Thus, the amino acid is considered the transportable form of N. Furthermore, although the source–sink amino acid regulation has been studied in other crops (Perchlik and Tegeder, 2017), little is known about cotton.

## 7 Functions of the key regulators involved in source–sink assimilate transport and distribution in cotton

The regulatory mechanisms underlying the source–sink relationships are complex and are affected by different factors, including hormones, signaling molecules, and assimilatory transport. Plant hormones play crucial roles in the regulation of source–sink relationships. Higher levels of cytokinin (CK) in leaves stimulate the Rubisco activity, thereby affecting the expression of photosynthetic genes and delaying leaf senescence (Cortleven and Schmuelling, 2015). Recent studies have shown that the auxin signaling cascade involving IAA-OsARF18-OsARF2 can regulate the allocation of sucrose sources and sinks, which, in turn, regulate the development of reproductive organs in rice (Zhao et al., 2022). Gibberellin (GA) is important for the regulation of plant growth and development, as well as biotic and abiotic stress responses (Xu et al., 2014; Hedden, 2020). DELLA protein is the negative regulator/primary growth inhibitor of GA signaling. GA promotes the nutritional and reproductive growth of plants by triggering the DELLA protein degradation (Xu et al., 2014). NITROGEN-MEDIATED TILLER GROWTH RESPONSE 5 (NGR5) was found to be an integrator of the auxin (IAA), strigolactone, and brassinosteroid (BR) signaling pathways (Aya et al., 2014; Hirano et al., 2017; Qiao et al., 2017; Fang et al., 2020). BRs promote N uptake by regulating the expression of NRT genes in maize (Xing et al., 2022). GROWTH-REGULATING FACTOR 4 (GRF4) promotes and integrates nitrogen assimilation, carbon fixation, and growth, whereas DELLA protein inhibits these processes. Balanced opposite action and physical interaction of the GRF4 transcription factor and the growth inhibitor DELLA synergistically regulates the growth and the carbon and nitrogen metabolism (Li et al., 2018a). The GRF4-DELLA-NGR5 modules interact to improve the nitrogen use efficiency (NUE), coordinate the source–sink relationships, and increase crop yield to achieve agricultural sustainability (Wu et al., 2021). Furthermore, ethylene (ETH) (Wang et al., 2012), abscisic acid (ABA) (Yang et al., 2001), and CK (Yang et al., 2000) also greatly affect the rice grain filling process. The plant growth regulator ethephon promotes the transport of stored and newly synthesized assimilates from the cotton leaves to the cotton boll, which can accumulate more in the cotton lint (Shen et al., 1980). Additionally, the external application of indoleacetic acid (IAA) promotes the activity of key sucrose metabolism-related enzymes in the cotton boll, embryo, and seed coat, thereby facilitating seed development (Yin et al., 2021). Although the influence of plant hormones on source-to-sink assimilate transport is similar among other crops, almost no study examined this in cotton.

Besides plant hormones, a few signaling molecules also act as key carbon and nitrogen metabolism regulators, thereby regulating the source–sink relationships. For example, ELONGATED HYPOCOTYL5 (HY5), which was one of the first light-responsive transcription factors to be identified, regulates the C transport in plant leaves, N uptake in roots, and root growth to balance the C/N levels in *Arabidopsis* (Chen et al., 2016). Additionally, the C and N substrates have also been found to act as signals. Although Trehalose 6-phosphate (T6P), a substrate in carbon metabolism, is present only in trace amounts, it possibly interacts with other sugar-signaling proteins, including hexokinase, 14-3-3 proteins, and SUCROSE NONFERMENTING-1-RELATED PROTEIN KINASE1 (SnRK1) to modulate the partitioning of photosynthetic products between sources and sinks, thereby affecting plant growth and development (Paul et al., 2008; Figueroa and Lunn, 2016). Nuccio et al. (2015) showed that expressing the rice *TREHALOSE-6-PHOSPHATE PHOSPHATASE* (*TPP*) gene in maize ears increased the sucrose concentration, kernel yield, and harvest index. Sucrose and hexose regulate carbon metabolism in the source organs (Koch, 1996) and cell expansion and division in sink organs (Bihmidine et al., 2013) similarly. SUGAR PARTITIONING AFFECTING (SPA) protein is a DnaJ molecular chaperone that regulates the distribution of glycans in plants. Bermudez et al. (2014) showed that inhibiting the *SPA* gene expression promotes the transport of photosynthetic products, like sucrose, fructose, and glucose from the leaves to sink; i.e., it regulates the source–sink relationship by modulating the transport of photosynthetic products to the fruits, which significantly increases the fruit quality.

Transport and partitioning of the carbon assimilate (usually sucrose) and nitrogen assimilate are also important for regulating the source–sink relationships in cotton (Minchin and Lacointe, 2005). Long-distance transport of sucrose from the source leaves to the sink organs occurs *via* leaf veins (Lambers and Oliveira, 2019). Numerous studies have functionally identified the key regulators involved in the assimilate partitioning in different crops (Bezrutczyk et al., 2018; Lu et al., 2020; Huang et al., 2021); however, there are relatively few publications on cotton plants, as presented in **Table 1**. As mentioned earlier, SUTs are critical for sucrose transport and distribution. The defective SUT can significantly affect the growth of cotton plants. *GhSUT2* and *GhSUT11* are expressed in both the source and sink organs, whereas *GhSUT6* and *GhSUT15* are mainly expressed in the sink organs (Chao et al., 2020). Furthermore, *GhSUT9* and *GhSUT18* are mainly expressed in the fibers (Chao et al., 2020). Studies showed that the *GhSWEET* gene may be involved in the developmental processes and different stress responses in cotton (Li et al., 2018b). The R2R3-MYB transcription factor, GhMYB212, regulates sucrose transport to the cotton fibers by regulating the expression of the sucrose transporter gene *GhSWEET12* (Sun et al., 2019). Based on the previous findings on the significance of *ZmSUT1* in maize sucrose allocation (Baker et al., 2016), Ding et al. (2019) investigated the impact of its expression on sucrose distribution in cotton. It was expressed in cotton under two different promoters: senescence-inducible promoter *PSAG12* and seed coat-specific promoter *BAN*. The *BAN*::*ZmSUT1* cotton exhibited increased photosynthetic rate, and improved sugar deposition in the boll and fiber, thereby indicating the potential roles of ZmSUT1 in promoting fiber yield by reprogramming sugar partitioning (Ding et al., 2019). To further investigate how partitioning of assimilates impacts the fiber growth and seed yield, Yadav et al. (2022) identified nine *SUT* gene family members, which are critical for assimilate partitioning in cotton. Among them, G*hSUT1-L2*, expressed in the mature leaves, was reported to promote carbohydrate accumulation in the leaves when silenced, thereby disrupting phloem transport. Therefore, understanding the mechanisms underlying the phloem loading and unloading in cotton may be a promising strategy for improving the assimilate partitioning to the sink organ (bolls, flowers, and fibers).

**Table 1:** Key regulators involved in the assimilate transport and distribution in cotton plants.

C/N	Genes	Transgenic approach	Summary of findings	Ref.
1	*GhMYB212*	RNA interference	Reduced sucrose in the developing fiber; Lower lint index and shorter fiber.	(Sun et al., 2019)
2	Potato *SUS*	Expression	Increased fructose concentration in young leaves and fiber; Enhanced leaf and seed development; Promoted fiber production.	(Xu et al., 2012)
3	*ZmSUT1*	Ectopic expression	Increased sucrose deposition in the fibers and bolls; Promoted cotton fiber and seed yield.	(Ding et al., 2019)
4	*GhSUT1-L2*	Gene silencing	Increased carbohydrate accumulation in the leaves; Disrupted phloem transport of sucrose.	(Yadav et al., 2022)
5	*GhSUT1*	Expression	Promoted sucrose accumulation; Enhanced fiber elongation at late developmental stages.	(Ruan et al., 2001; Zhang et al., 2016b; Guo et al., 2017)
6	*GhSUT9*	Expression	Increased sucrose and starch content in the anther	(Yu et al., 2023)

## 8 Concluding remarks and prospects

Along with the increased source capacity, the efficient assimilate allocation to the sink is most promising for enhancing the seed and fiber yield. However, despite much effort in exploring the source–sink relationships, studies have not elucidated the regulatory mechanisms of source–sink relationships. Boll (sink) and mature-leaf (source) in cotton are the most prominent plant organs, which house the photosynthetic assimilates. The nutrient distribution between boll and leaf reflects the coordination between nutrient growth and reproductive growth in cotton plants, which affects the yield and quality of cotton. Understanding how photosynthetic products, nitrogen, etc. are transported from the source leaves to the sinks is imperative. The precise identification of genes related to the transport and distribution of carbon assimilates (e.g., sucrose) and nitrogen assimilates (e.g., nitrate) will provide an in-depth understanding of the regulatory mechanisms underlying the source–sink relationships in cotton and thus help in breeding high-yielding cotton. As shown in **Figure 2**, this review provides insight into the schematic direction of deciphering the regulation mechanism of the source–sink relationship in cotton. Therefore, we expect that the current review might help future researchers to design the corresponding research strategy.

The underlying regulatory mechanisms of the source–sink relationship in crop plants are complex. This requires first screening and identifying the key genes and metabolites, followed by verifying their functions to decode and manipulate the regulatory network. In recent years, the emerging single-cell RNA sequencing technology has been successfully utilized in plants to study tissues and cells and detect genes at the single-cell resolution. However, due to the complexities in plant tissue structure, challenges of tissue permeability can limit the application of this technique in plants (Liu et al., 2020). Furthermore, several other technologies including gas chromatography-mass spectrometry (GC-MS) (Naik et al., 2021), desorption electrospray ionization mass spectrometry imaging (DESI-MSI) (Takáts et al., 2004), matrix-assisted laser desorption/ionization mass spectrometry imaging (MALDI-MSI) (Stoeckli et al., 2001), and air flow-assisted desorption electrospray ionization mass spectrometry imaging (AFADESI-MSI) (He et al., 2018) have been used to detect the key metabolites in plant research. These metabolite-detector techniques have higher sensitivity and allow more comprehensive detection and spatial imaging of the metabolites. Although these techniques have their strengths and limitations, further improvement strategies are needed to tackle the limitations hindering the detection of sucrose-related genes and metabolite in plants.

The application of the source–sink relationship in crop production is also an important open question currently being addressed by the research and breeding community. It is well known that the photosynthetic efficiency of plants is closely related to yield. With the rapid development of synthetic biology and other disciplines, researchers have recently proposed to improve redesign light coordination to improve crop yields (Ort et al., 2015; Zhu et al., 2022). The proposed areas for photosynthetic improvement include, but are not limited to the following:

- Structure and molecular regulation of transporters and enzymes involved in photosynthesis
- Variation of gene regulatory networks reprogramming photosynthesis
- Factors regulating stomatal conductance
- Factors influencing leaf and canopy photosynthesis
- Photosynthesis under fluctuating climate or stressed conditions
- Recent advances in natural genetic variation, especially in cotton plants
- The use of molecular biology approach to better understand enzymes and pathways involved in photosynthesis

The Clustered Regularly Interspaced Short Palindromic Repeats (CRISPR)-associated protein 9 (CRISPR/Cas 9) system has been widely used in plant genome editing due to its several advantages, like low cost, simplicity, and efficiency, especially for multiple genome editing and regulation of gene expression (Cong et al., 2013; Yin et al., 2017). The introduction of the CRISPR/Cas 9 system for cotton has helped to solve a variety of yield-related problems and improve fibre development (Zhou et al., 2015; Li et al., 2017; Peng et al., 2021). CRISPR/Cas 9 technology has the potential to harness various genetic cascades of traits while addressing breeding-related issues in cotton (Sattar et al., 2019; Fiaz et al., 2021). Beyond its use in specific gene knockout (loss of function), CRISPR/Cas9 knocks in and repairs individual genes at the transcriptional level (Ahmad et al., 2020; Bukhari et al., 2021). Another vital application of this genome editing in fiber production is to improve cotton tolerance to abiotic stress (Wang et al., 2017). Despite these glamorous prospects, difficulties in regenerating plant have been the major bottleneck with the use of CRISPR/Cas 9 genome editing in cotton. Most research has employed the agrobacterium-mediated method of transformation and gene editing (Ramadan et al., 2021). This method, however, proves abortive because of the need for tissue culture and cotton regeneration, thus limiting the application of CRISPR/Cas 9 genome editing in cotton. To improve the regeneration capacity, Peng et al. (2021) proposed the need for germplasm with a high frequency of cotton somatic embryogenesis. With that, a highly efficient regeneration system that can promote the use of CRISPR/Cas 9 genome editing in cotton will be established.

To enhance crop yield, increasing the source or sink strength is not enough; the source and sink intensities must be coordinated and enhanced simultaneously. Therefore, we propose a possible solution to improve crop yield by regulating and improving the source–sink relationship through genetic approaches.

The following putative measures can be explored to achieve the improved crop yield: (1) optimizing the photosynthetic efficiency of the source, thereby promoting its activity and increasing the supply capacity of the source to the sink; (2) correspondingly, the appropriate assimilate transport genes can be overexpressed in specific tissues, like the phloem, to promote the assimilate transport from the source to the sink; and (3) finally, by overexpressing the genes specifically expressed at the end of the sink, its carrying capacity can be improved, which increases the storage of the sink by enhancing the assimilates. Therefore, studies on source–sink relationship in cotton seem promising, and further work on such related area could improve the cotton yield and quality. Multidisciplinary research is thus a prerequisite for applying the source–sink theory to improve future crop productivity.

## Author contributions

Conceptualization of the project: XS. Manuscript drafting: AQ, OA, ZL, JY, MH, and LG. Contribution to the editing and proofreading of the manuscript draft: ZL and XS. All authors contributed to the article and approved the submitted version.

## References

[B1] Ahmad N. Rahman M. U. Mukhtar Z. Zafar Y. Zhang B. (2020). A critical look on CRISPR-based genome editing in plants. J. Cell. Physiol. 235, 666–682. doi: 10.1002/jcp.29052 31317541

[B2] Ainsworth E. A. Long S. P. (2005). What have we learned from 15 years of free-air CO2 enrichment (FACE)? A meta-analytic review of the responses of photosynthesis, canopy properties and plant production to rising CO2. New Phytol. 165, 351–372. doi: 10.1111/j.1469-8137.2004.01224.x 15720649

[B3] Allen L. Kimball B. Bunce J. Yoshimoto M. Harazono Y. Baker J.. (2020). Fluctuations of CO2 in free-air CO2 enrichment (FACE) depress plant photosynthesis, growth, and yield. Agric. For. Meteorology 284, 107899. doi: 10.1016/j.agrformet.2020.107899

[B4] Aya K. Hobo T. Sato-Izawa K. Ueguchi-Tanaka M. Kitano H. Matsuoka M. (2014). A novel AP2-type transcription factor, SMALL ORGAN SIZE1, controls organ size downstream of an auxin signaling pathway. Plant Cell Physiol. 55, 897–912. doi: 10.1093/pcp/pcu023 24486766

[B5] Baker J. T. Lascano R. J. Yates C. Gitz Iii D. C. (2022). Nighttime CO2 enrichment did not increase leaf area or shoot biomass in cotton seedlings. Agric. For. Meteorology 320, 108931. doi: 10.1016/j.agrformet.2022.108931

[B6] Baker R. F. Leach K. A. Boyer N. R. Swyers M. J. Benitez-Alfonso Y. Skopelitis T.. (2016). Sucrose transporter ZmSut1 expression and localization uncover new insights into sucrose phloem loading. Plant Physiol. 172, 1876–1898. doi: 10.1104/pp.16.00884 27621426 PMC5100798

[B7] Benedict C. R. Kohel R. J. Schubert A. M. Keithly J. H. (1981). Species variation of photosynthesis in gossypium. Agronomy 7, 9–80.

[B8] Bermudez L. De Godoy F. Baldet P. Demarco D. Osorio S. Quadrana L.. (2014). Silencing of the tomato sugar partitioning affecting protein (SPA) modifies sink strength through a shift in leaf sugar metabolism. Plant J. 77, 676–687. doi: 10.1111/tpj.12418 24372694

[B9] Bezrutczyk M. Hartwig T. Horschman M. Char S. N. Yang J. Yang B.. (2018). Impaired phloem loading in zmsweet13a, b, c sucrose transporter triple knock-out mutants in zea mays. New Phytol. 218, 594–603. doi: 10.1111/nph.15021 29451311

[B10] Bhattacharya S. Kundu A. (2020). “Sugars and sugar polyols in overcoming environmental stresses,” in Protective chemical agents in the amelioration of plant abiotic stress: Biochemical and molecular perspectives, 71–101. doi: 10.1002/9781119552154.chapter4

[B11] Bihmidine S. Hunter C. T. 3rd Johns C. E. Koch K. E. Braun D. M. (2013). Regulation of assimilate import into sink organs: Update on molecular drivers of sink strength. Front. Plant Sci. 4, 177. doi: 10.3389/fpls.2013.00177 23761804 PMC3671192

[B12] Braun D. M. Wang L. Ruan Y. L. (2014). Understanding and manipulating sucrose phloem loading, unloading, metabolism, and signalling to enhance crop yield and food security. J. Exp. Bot. 65, 1713–1735. doi: 10.1093/jxb/ert416 24347463

[B13] Bukhari S. A. R. Saeed M. Briddon R. W. (2021). “Use of CRISPR/Cas system to create resistance to cotton diseases. In Rahman M.-U. Zafar Y. Zhang T. (Eds.), Cotton Precision Breeding (pp. 329–350). Springer International Publishing. doi: 10.1007/978-3-030-64504-5_15

[B14] Chao M. Wang B. Chen Y. Zhang J. Sun X. Wang Q. (2020). Identification and expression analysis of sucrose transporter gene family in upland cotton (Gossypium hirsutum l.). Acta Botanica Boreali-Occidentalia Sin. 40, 1303–1312. (In Chinese)

[B15] Chen Y. Li Y. B. Zhou M. Y. Rui Q. Z. Cai Z. Z. Zhang X.. (2018). Nitrogen (N) application gradually enhances boll development and decreases boll shell insecticidal protein content in n-deficient cotton. Front. Plant Sci. 9. doi: 10.3389/fpls.2018.00051 PMC5797636 29441082

[B16] Chen J. Liu L. T. Wang Z. B. Zhang Y. J. Sun H. C. Song S. J.. (2020). Nitrogen fertilization increases root growth and coordinates the root-shoot relationship in cotton. Front. Plant Sci. 11. doi: 10.3389/fpls.2020.00880 PMC7324761 32655605

[B17] Chen L.-Q. Qu X.-Q. Hou B.-H. Sosso D. Osorio S. Fernie A. R.. (2012a). Sucrose efflux mediated by SWEET proteins as a key step for phloem transport. Science 335, 207–211. doi: 10.1126/science.1213351 22157085

[B18] Chen Q. Yang G. Zhang X. Nie Y. (2012b). Review on nitrogen nutrition characteristic of cotton. Chin. Agric. Sci. Bull. 28, 15–19. (in chinese)

[B19] Chen X. Yao Q. Gao X. Jiang C. Harberd N. P. Fu X. (2016). Shoot-to-Root mobile transcription factor HY5 coordinates plant carbon and nitrogen acquisition. Curr. Biol. 26, 640–646. doi: 10.1016/j.cub.2015.12.066 26877080

[B20] Clement J. D. Constable G. A. Conaty W. C. (2013). CO2 exchange rate in cotton does not explain negative associations between lint yield and fiber quality. J. Cotton Sci. 17, 270–278.

[B21] Cong L. Ran F. A. Cox D. Lin S. L. Barretto R. Habib N.. (2013). Multiplex genome engineering using CRISPR/Cas systems. Science 339, 819–823. doi: 10.1126/science.1231143 23287718 PMC3795411

[B22] Cornish K. Radin J. W. Turcotte E. L. Lu Z. Zeiger E. (1991). Enhanced photosynthesis and stomatal conductance of pima cotton (Gossypium barbadense l.) bred for increased yield. Plant Physiol. 97, 484–489. doi: 10.1104/pp.97.2.484 16668424 PMC1081032

[B23] Cortleven A. Schmuelling T. (2015). Regulation of chloroplast development and function by cytokinin. J. Exp. Bot. 66, 4999–5013. doi: 10.1093/jxb/erv132 25873684

[B24] Cui G. Zhang Y. Zhang W. Lang D. Zhang X. Li Z.. (2019). Response of carbon and nitrogen metabolism and secondary metabolites to drought stress and salt stress in plants. J. Plant Biol. 62, 387–399. doi: 10.1007/s12374-019-0257-1

[B25] Dai J. Luo Z. Li W. Tang W. Zhang D. Lu H.. (2014). A simplified pruning method for profitable cotton production in the yellow river valley of China. Field Crops Res. 164, 22–29. doi: 10.1016/j.fcr.2014.05.010

[B26] Ding X. Zeng J. Huang L. Li X. Song S. Pei Y. (2019). Senescence-induced expression of ZmSUT1 in cotton delays leaf senescence while the seed coat-specific expression increases yield. Plant Cell Rep. 38, 991–1000. doi: 10.1007/s00299-019-02421-1 31069498

[B27] Dong H. Yang G. Li Y. Tian L. Kong X. (2017). Key technologies for light and simplified cultivation of cotton and their eco-physiological mechanisms. Acta Agronomica Sin. 43, 631–639. doi: 10.3724/SP.J.1006.2017.00631

[B28] Dong H. Zhang D. Tang W. Li W. Li Z. (2005). Effects of planting system, plant density and flower removal on yield and quality of hybrid seed in cotton. Field Crops Res. 93, 74–84. doi: 10.1016/j.fcr.2004.09.010

[B29] Du Y. Zhao Q. Chen L. Yao X. Zhang H. Wu J.. (2020). Effect of drought stress during soybean R2–R6 growth stages on sucrose metabolism in leaf and seed. Int. J. Mol. Sci. 21, 618. doi: 10.3390/ijms21020618 31963537 PMC7013680

[B30] Elmore C. Hesketh J. Muramoto H. (1967). A survey of rates of leaf growth, leaf aging and leaf photosynthetic rates among and within species. J. Arizona Acad. Sci. 4, 215–219. doi: 10.2307/40022411

[B31] Fang Z. Ji Y. Hu J. Guo R. Sun S. Wang X. (2020). Strigolactones and brassinosteroids antagonistically regulate the stability of the D53-OsBZR1 complex to determine FC1 expression in rice tillering. Mol. Plant 13, 586–597. doi: 10.1016/j.molp.2019.12.005 31837469

[B32] Faralli M. Lawson T. (2020). Natural genetic variation in photosynthesis: an untapped resource to increase crop yield potential? Plant J. 101, 518–528. doi: 10.1111/tpj.14568 31625637 PMC7028090

[B33] Fiaz S. Khan S. A. Younas A. Shahzad K. Ali H. Noor M. A.. (2021). Chapter 13 - Application of CRISPR/Cas system for genome editing in cotton. In Abd-Elsalam K. A. Lim K.-T. (Eds.), CRISPR and RNAi systems (Elsevier), 277–301. doi: 10.1016/B978-0-12-821910-2.00010-2

[B34] Figueroa C. M. Lunn J. E. (2016). A tale of two sugars: Trehalose 6-phosphate and Sucrose(1 OPEN ). Plant Physiol. 172, 7–27. doi: 10.1104/pp.16.00417 27482078 PMC5074632

[B35] Fritschi F. B. Roberts B. A. Travis R. L. Rains D. W. Hutmacher R. B. (2003). Response of irrigated acala and pima cotton to nitrogen fertilization: Growth, dry matter partitioning, and yield. Agron. J. 95, 133–146. doi: 10.2134/agronj2003.1330a

[B36] Gao M. Snider J. L. Bai H. Hu W. Wang R. Meng Y.. (2020). Drought effects on cotton (Gossypium hirsutum l.) fibre quality and fibre sucrose metabolism during the flowering and boll-formation period. J. Agron. Crop Sci. 206, 309–321. doi: 10.1111/jac.12389

[B37] Gaxiola R. A. Palmgren M. G. Schumacher K. (2007). Plant proton pumps. FEBS Lett. 581, 2204–2214. doi: 10.1016/j.febslet.2007.03.050 17412324

[B38] Guo Y. Song H. Zhao Y. Qin X. Cao Y. Zhang L. (2021). Switch from symplasmic to aspoplasmic phloem unloading in xanthoceras sorbifolia fruit and sucrose influx XsSWEET10 as a key candidate for sugar transport. Plant Sci. 313, 111089. doi: 10.1016/j.plantsci.2021.111089 34763874

[B39] Guo K. Tu L. He Y. Deng J. Wang M. Huang H.. (2017). Interaction between calcium and potassium modulates elongation rate in cotton fiber cells. J. Exp. Bot. 68, 5161–5175. doi: 10.1093/jxb/erx346 29045717 PMC5853336

[B40] Han X. (2015). New crop breeding technique from source-path-sink theory. Biotechnol. Bull. 31, 34–39. doi: 10.13560/j.cnki.biotech.bull.1985.2015.03.019

[B41] Harischandra N. R. Pallavi M. S. Bheemanna M. Pavankumar K. Chandra Sekhara Reddy V. Udaykumar N. R.. (2021). Simultaneous determination of 79 pesticides in pigeonpea grains using GC-MS/MS and LC-MS/MS. Food Chem. 347, 128986. doi: 10.1016/j.foodchem.2020.128986 33515969

[B42] He Q. Dong S. Gao R. (2005). Relationship between development of spike vascular bundle and sink capacity of ear and kernel in maize (Zea mays l.). Acta Agronomica Sin. 31, 995–1000.

[B43] He J. M. Sun C. L. Li T. G. Luo Z. G. Huang L. J. Song X. W.. (2018). A sensitive and wide coverage ambient mass spectrometry imaging method for functional metabolites based molecular histology. Adv. Sci. (Weinh) 5(11), 1800250. doi: 10.1002/advs.201800250. Erratum in: Adv Sci (Weinh). 2019 Jan 09;6(1):1802201 30643733 PMC6325592

[B44] Hedden P. (2020). The current status of research on gibberellin biosynthesis. Plant Cell Physiol. 61, 1832–1849. doi: 10.1093/pcp/pcaa092 32652020 PMC7758035

[B45] Hezhong D. Weijiang L. Wei T. (2007). Effects of retention of vegetative branches on source-sink relation, leaf senescence and lint yield in bt transgenic hybrid cotton. Scientia Agricultura Sinica.

[B46] Hirano K. Yoshida H. Aya K. Kawamura M. Hayashi M. Hobo T.. (2017). SMALL ORGAN SIZE 1 and SMALL ORGAN SIZE 2/DWARF AND LOW-TILLERING form a complex to integrate auxin and brassinosteroid signaling in rice. Mol. Plant 10, 590–604. doi: 10.1016/j.molp.2016.12.013 28069545

[B47] Huang X. Zhang Y. Wang L. Dong X. Hu W. Jiang M.. (2021). OsDOF11 affects nitrogen metabolism by sucrose transport signaling in rice (Oryza sativa l.). Front. Plant Sci. 12, 1788. doi: 10.3389/fpls.2021.703034 PMC8461328 34567026

[B48] Huppe H. C. Turpin D. H. (1994). Integration of carbon and nitrogen metabolism in plant and algal cells. Annu. Rev. Plant Biol. 45, 577–607. doi: 10.1146/annurev.pp.45.060194.003045

[B49] Iqbal A. Dong Q. Wang X. Gui H. Zhang H. Zhang X.. (2020). Transcriptome analysis reveals differences in key genes and pathways regulating carbon and nitrogen metabolism in cotton genotypes under n starvation and resupply. Int. J. Mol. Sci. 21, 1500. doi: 10.3390/ijms21041500 32098345 PMC7073098

[B50] Jin P. Wu D. Dai H. Sun R. Liu A. (2022). Characterization and functional divergence of genes encoding sucrose transporters in oilseeds castor bean. Oil Crop Sci. 7, 31–39. doi: 10.1016/j.ocsci.2022.02.003

[B51] Kimball B. Pinter P. Jr. Wall G. W. Garcia R. Lamorte R. Jak P. M.. (1997). Comparisons of responses of vegetation to elevated carbon dioxide in free-air and open-top chamber facilities. In Advances in Carbon Dioxide Effects Research, 113–130. doi: 10.2134/asaspecpub61.c5

[B52] Ko H.-Y. Ho L.-H. Neuhaus H. E. Guo W.-J. (2021). Transporter SlSWEET15 unloads sucrose from phloem and seed coat for fruit and seed development in tomato. Plant Physiol. 187, 2230–2245. doi: 10.1093/plphys/kiab290 34618023 PMC8644451

[B53] Koch K. E. (1996). Carbohydrate-modulated gene expression in plants. Annu. Rev. Plant Physiol. Plant Mol. Biol. 47, 509–540. doi: 10.1146/annurev.arplant.47.1.509 15012299

[B54] Lambers H. Oliveira R. S. (2019). “Photosynthesis, respiration, and long-distance transport: Long distance transport of assimilates,” in Plant physiological ecology (Cham: Springer International Publishing), 173–186. doi: 10.1007/978-3-030-29639-1_2

[B55] Lei Z. Liu F. Wright I. J. Carriquí M. Niinemets Ü. Han J.. (2022). Comparisons of photosynthetic and anatomical traits between wild and domesticated cotton. J. Exp. Bot. 73, 873–885. doi: 10.1093/jxb/erab293 34153103

[B56] Lemoine R. La Camera S. Atanassova R. Dedaldechamp F. Allario T. Pourtau N.. (2013). Source-to-sink transport of sugar and regulation by environmental factors. Front. Plant Sci. 4. doi: 10.3389/fpls.2013.00272 PMC3721551 23898339

[B57] Li W. Ren Z. Wang Z. Sun K. Pei X. Liu Y.. (2018b). Evolution and stress responses of gossypium hirsutum SWEET genes. Int. J. Mol. Sci. 19 (3), 769. doi: 10.3390/ijms19030769 29517986 PMC5877630

[B58] Li S. Tian Y. H. Wu K. Ye Y. F. Yu J. P. Zhang J. Q.. (2018a). Modulating plant growth-metabolism coordination for sustainable agriculture. Nature 560, 595–59+. doi: 10.1038/s41586-018-0415-5 30111841 PMC6155485

[B59] Li C. Unver T. Zhang B. (2017). A high-efficiency CRISPR/Cas9 system for targeted mutagenesis in cotton (Gossypium hirsutum l.). Sci. Rep. 7, 1–10. doi: 10.1038/srep43902 28256588 PMC5335549

[B60] Li N. Yao N. Li Y. Chen J. Liu D. Biswas A.. (2021). A meta-analysis of the possible impact of climate change on global cotton yield based on crop simulation approaches. Agric. Syst. 193, 103221. doi: 10.1016/j.agsy.2021.103221

[B61] Liu J. Meng Y. Lv F. Chen J. Ma Y. Wang Y.. (2015). Photosynthetic characteristics of the subtending leaf of cotton boll at different fruiting branch nodes and their relationships with lint yield and fiber quality. Front. Plant Sci. 6, 747. doi: 10.3389/fpls.2015.00747 26442060 PMC4584985

[B62] Liu Z. Zhou Y. Guo J. Li J. Tian Z. Zhu Z.. (2020). Global dynamic molecular profiling of stomatal lineage cell development by single-cell RNA sequencing. Mol. Plant 13, 1178–1193. doi: 10.1016/j.molp.2020.06.010 32592820

[B63] Loka D. A. Oosterhuis D. M. Baxevanos D. Noulas C. Hu W. (2020). Single and combined effects of heat and water stress and recovery on cotton (Gossypium hirsutum l.) leaf physiology and sucrose metabolism. Plant Physiol. Biochem. 148, 166–179. doi: 10.1016/j.plaphy.2020.01.015 31962205

[B64] Lu M. Z. Snyder R. Grant J. Tegeder M. (2020). Manipulation of sucrose phloem and embryo loading affects pea leaf metabolism, carbon and nitrogen partitioning to sinks as well as seed storage pools. Plant J. 101, 217–236. doi: 10.1111/tpj.14533 31520495

[B65] Mangi N. Nazir M. F. Wang X. Iqbal M. S. Sarfraz Z. Jatoi G. H.. (2021). Dissecting source-sink relationship of subtending leaf for yield and fiber quality attributes in upland cotton (Gossypium hirsutum l.). Plants 10, 1147. doi: 10.3390/plants10061147 34199872 PMC8229918

[B66] Mason T. G. Maskell E. J. (1928). Studies on the transport of carbohydrates in the cotton plant: II. the factors determining the rate and the direction of movement of sugars. Ann. Bot. 42, 571–636. doi: 10.1093/oxfordjournals.aob.a090131

[B67] McGarry R. C. Prewitt S. F. Culpepper S. Eshed Y. Lifschitz E. Ayre B. G. (2016). Monopodial and sympodial branching architecture in cotton is differentially regulated by the gossypium hirsutum SINGLE FLOWER TRUSS and SELF-PRUNING orthologs. New Phytol. 212, 244–258. doi: 10.1111/nph.14037 27292411

[B68] Minchin P. E. H. Lacointe A. (2005). New understanding on phloem physiology and possible consequences for modelling long-distance carbon transport. New Phytol. 166, 771–779. doi: 10.1111/j.1469-8137.2005.01323.x 15869640

[B69] Mollaee M. Mobli A. Mutti N. K. Manalil S. Chauhan B. S. (2019). Challenges and opportunities in cotton production. cotton production, 371–390. doi: 10.1002/9781119385523.ch18

[B70] Morgan J. A. Lecain D. R. Mosier A. R. Milchunas D. G. (2001). Elevated CO2 enhances water relations and productivity and affects gas exchange in C3 and C4 grasses of the Colorado shortgrass steppe. Global Change Biol. 7, 451–466. doi: 10.1046/j.1365-2486.2001.00415.x

[B71] Naikwade P. (2020). Effect of climate change on growth and productivity of cotton: Global scenario. The International Journal of Analytical and Experimental Modal Analysis. 12, 64.

[B72] Nie J. Li Z. Zhang Y. Zhang D. Xu S. He N.. (2021). Plant pruning affects photosynthesis and photoassimilate partitioning in relation to the yield formation of field-grown cotton. Ind. Crops Products 173, 114087. doi: 10.1016/j.indcrop.2021.114087

[B73] Nie J. Qin D. Mao L. Liu Y. Dong H. Song X.. (2020). Genotypic variance in 13C-photosynthate partitioning and within-plant boll distribution in cotton. J. Cotton Res. 3, 1–10. doi: 10.1186/s42397-020-00055-3

[B74] Nuccio M. L. Wu J. Mowers R. Zhou H.-P. Meghji M. Primavesi L. F.. (2015). Expression of trehalose-6-phosphate phosphatase in maize ears improves yield in well-watered and drought conditions. Nat. Biotechnol. 33, 862–86+. doi: 10.1038/nbt.3277 26473199

[B75] Ort D. R. Merchant S. S. Alric J. Barkan A. Blankenship R. E. Bock R.. (2015). Redesigning photosynthesis to sustainably meet global food and bioenergy demand. Proc. Natl. Acad. Sci. United States America 112, 8529–8536. doi: 10.1073/pnas.1424031112 PMC4507207 26124102

[B76] Paul M. J. Primavesi L. F. Jhurreea D. Zhang Y. (2008). Trehalose metabolism and signaling. Annu. Rev. Plant Biol. 59, 417–441. doi: 10.1146/annurev.arplant.59.032607.092945 18257709

[B77] Peng R. Jones D. C. Liu F. Zhang B. (2021). From sequencing to genome editing for cotton improvement. Trends Biotechnol. 39, 221–224. doi: 10.1016/j.tibtech.2020.09.001 32988631

[B78] Perchlik M. Tegeder M. (2017). Improving plant nitrogen use efficiency through alteration of amino acid transport processes. Plant Physiol. 175, 235–247. doi: 10.1104/pp.17.00608 28733388 PMC5580756

[B79] Pettigrew W. Gerik T. (2007). Cotton leaf photosynthesis and carbon metabolism. Adv. Agron. 94, 209–236. doi: 10.1016/S0065-2113(06)94005-X

[B80] Pettigrew W. Turley R. (1998). Variation in photosynthetic components among photosynthetically diverse cotton genotypes. Photosynthesis Res. 56, 15–25. doi: 10.1023/A:1005902028459

[B81] Pilkington S. M. Encke B. Krohn N. Hoehne M. Stitt M. Pyl E. T. (2015). Relationship between starch degradation and carbon demand for maintenance and growth in a rabidopsis thaliana in different irradiance and temperature regimes. Plant Cell Environ. 38, 157–171. doi: 10.1111/pce.12381 24905937

[B82] Pilon C. Loka D. Snider J. L. Oosterhuis D. M. (2019). Drought-induced osmotic adjustment and changes in carbohydrate distribution in leaves and flowers of cotton (Gossypium hirsutum l.). J. Agron. Crop Sci. 205, 168–178. doi: 10.1111/jac.12315

[B83] Prentice I. C. Farquhar G. Fasham M. Goulden M. L. Heimann M. Jaramillo V.. (2001). The carbon cycle and atmospheric carbon dioxide. Climate Change 2001: The Scientific Basis, Intergovernmental Panel on Climate Change, 2001. hal-03333974.

[B84] Qiao S. Sun S. Wang L. Wu Z. Li C. Li X.. (2017). The RLA1/SMOS1 transcription factor functions with OsBZR1 to regulate brassinosteroid signaling and rice architecture. Plant Cell 29, 292–309. doi: 10.1105/tpc.16.00611 28100707 PMC5354187

[B85] Ramadan M. Alariqi M. Ma Y. Li Y. Liu Z. Zhang R.. (2021). Efficient CRISPR/Cas9 mediated pooled-sgRNAs assembly accelerates targeting multiple genes related to male sterility in cotton. Plant Methods 17, 1–13. doi: 10.1186/s13007-021-00712-x 33557889 PMC7869495

[B86] Ruan Y. L. Llewellyn D. J. Furbank R. T. (2001). The control of single-celled cotton fiber elongation by developmentally reversible gating of plasmodesmata and coordinated expression of sucrose and k+ transporters and expansin. Plant Cell 13, 47–60. doi: 10.1105/tpc.13.1.47 11158528 PMC102212

[B87] Sattar M. N. Iqbal Z. Dangol S. D. Bakhsh A. (2019). “CRISPR/Cas9: A new genome editing tool to accelerate cotton (Gossypium spp.) breeding. In: Al-Khayri J. Jain S. Johnson D. (eds) Advances in Plant Breeding Strategies: Industrial and Food Crops. (Cham: Springer). doi: 10.1007/978-3-030-23265-8_3

[B88] Shah A. N. Iqbal J. Tanveer M. Yang G. Hassan W. Fahad S.. (2017). Nitrogen fertilization and conservation tillage: A review on growth, yield, and greenhouse gas emissions in cotton. Environ. Sci. pollut. Res. 24, 2261–2272. doi: 10.1007/s11356-016-7894-4 27796993

[B89] Shareef M. Gui D. Zeng F. Ahmed Z. Waqas M. Zhang B.. (2018). Impact of drought on assimilates partitioning associated fruiting physiognomies and yield quality attributes of desert grown cotton. Acta Physiologiae Plantarum 40, 1–12. doi: 10.1007/s11738-018-2646-3

[B90] Shen Y. Q. Fang B. C. Sheng M. Z. (1980). The effect of photosynthesis and translocation in cotton leaf. J. Integr. Plant Biol. 2, 136–140. Available at: https://www.jipb.net/CN/abstract/article_24554.shtml

[B91] Sonnewald U. Fernie A. R. (2018). Next-generation strategies for understanding and influencing source-sink relations in crop plants. Curr. Opin. Plant Biol. 43, 63–70. doi: 10.1016/j.pbi.2018.01.004 29428477

[B92] Stitt M. Muller C. Matt P. Gibon Y. Carillo P. Morcuende R.. (2002). Steps towards an integrated view of nitrogen metabolism. J. Exp. Bot. 53, 959–970. doi: 10.1093/jexbot/53.370.959 11912238

[B93] Stoeckli M. Chaurand P. Hallahan D. E. Caprioli R. M. (2001). Imaging mass spectrometry: A new technology for the analysis of protein expression in mammalian tissues. Nat. Med. 7, 493–496. doi: 10.1038/86573 11283679

[B94] Sun W. Gao Z. Wang J. Huang Y. Chen Y. Li J.. (2019). Cotton fiber elongation requires the transcription factor GhMYB212 to regulate sucrose transportation into expanding fibers. New Phytol. 222, 864–881. doi: 10.1111/nph.15620 30506685

[B95] Takáts Z. Wiseman J. M. Gologan B. Cooks R. G. (2004). Mass spectrometry sampling under ambient conditions with desorption electrospray ionization. Science 306, 471–473. doi: 10.1126/science.1104404 15486296

[B96] Tang F. Zhu J. Wang T. Shao D. (2017). Water deficit effects on carbon metabolism in cotton fibers during fiber elongation phase. Acta physiologiae plantarum 39, 1–9. doi: 10.1007/s11738-017-2368-y

[B97] Tegeder M. Masclaux-Daubresse C. (2018). Source and sink mechanisms of nitrogen transport and use. New Phytol. 217, 35–53. doi: 10.1111/nph.14876 29120059

[B98] Ul-Allah S. Rehman A. Hussain M. Farooq M. (2021). Fiber yield and quality in cotton under drought: Effects and management. Agric. Water Manage. 255, 106994. doi: 10.1016/j.agwat.2021.106994

[B99] Van Bel A. J. (2021). The plant axis as the command centre for (re) distribution of sucrose and amino acids. J. Plant Physiol. 265, 153488. doi: 10.1016/j.jplph.2021.153488 34416599

[B100] Wang R. Ji S. Zhang P. Meng Y. Wang Y. Chen B.. (2016). Drought effects on cotton yield and fiber quality on different fruiting branches. Crop Sci. 56, 1265–1276. doi: 10.2135/cropsci2015.08.0477

[B101] Wang Y. Meng Z. Liang C. Meng Z. Wang Y. Sun G.. (2017). Increased lateral root formation by CRISPR/Cas9-mediated editing of arginase genes in cotton. Sci. China. Life Sci. 60, 524. doi: 10.1007/s11427-017-9031-y 28527115

[B102] Wang Z. Xu Y. Wang J. Yang J. Zhang J. (2012). Polyamine and ethylene interactions in grain filling of superior and inferior spikelets of rice. Plant Growth Regul. 66, 215–228. doi: 10.1007/s10725-011-9644-4

[B103] White A. C. Rogers A. Rees M. Osborne C. P. (2016). How can we make plants grow faster? A source-sink perspective on growth rate. J. Exp. Bot. 67, 31–45. doi: 10.1093/jxb/erv447 26466662

[B104] Wu K. Xu H. Gao X. H. Fu X. D. (2021). New insights into gibberellin signaling in regulating plant growth-metabolic coordination. Curr. Opin. Plant Biol. 63, 102074. doi: 10.1016/j.pbi.2021.102074 34217918

[B105] Xing F. Han Y. Feng L. Zhi X. Wang G. Yang B.. (2018). Genotypic variation in spatiotemporal distribution of canopy light interception in relation to yield formation in cotton. J. Cotton Res. 1, 1–10. doi: 10.1186/s42397-018-0012-z

[B106] Xing J. P. Wang Y. B. Yao Q. Q. Zhang Y. S. Zhang M. C. Li Z. H. (2022). Brassinosteroids modulate nitrogen physiological response and promote nitrogen uptake in maize (Zea mays l.). Crop J. 10, 166–176. doi: 10.1016/j.cj.2021.04.004

[B107] Xu S.-M. Brill E. Llewellyn D. J. Furbank R. T. Ruan Y.-L. (2012). Overexpression of a potato sucrose synthase gene in cotton accelerates leaf expansion, reduces seed abortion, and enhances fiber production. Mol. Plant 5, 430–441. doi: 10.1093/mp/ssr090 22115917

[B108] Xu H. Liu Q. Yao T. Fu X. D. (2014). Shedding light on integrative GA signaling. Curr. Opin. Plant Biol. 21, 89–95. doi: 10.1016/j.pbi.2014.06.010 25061896

[B109] Yadav U. P. Evers J. F. Shaikh M. A. Ayre B. G. (2022). Cotton phloem loads from the apoplast using a single member of its nine-member sucrose transporter gene family. J. Exp. Bot. 73, 848–859. doi: 10.1093/jxb/erab461 34687198

[B110] Yang Y. Chen M. Tian J. Xiao F. Xu S. Zuo W.. (2019). Improved photosynthetic capacity during the mid-and late reproductive stages contributed to increased cotton yield across four breeding eras in xinjiang, China. Field Crops Res. 240, 177–184. doi: 10.1016/j.fcr.2018.11.003

[B111] Yang J. C. Peng S. B. Visperas R. M. Sanico A. L. Zhu Q. S. Gu S. L. (2000). Grain filling pattern and cytokinin content in the grains and roots of rice plants. Plant Growth Regul. 30, 261–270. doi: 10.1023/A:1006356125418

[B112] Yang J. C. Zhang J. H. Wang Z. Q. Zhu Q. S. Wang W. (2001). Hormonal changes in the grains of rice subjected to water stress during grain filling. Plant Physiol. 127, 315–323. doi: 10.1104/pp.127.1.315 11553759 PMC117987

[B113] Yao H. Zhang Y. Yi X. Zuo W. Lei Z. Sui L.. (2017). Characters in light-response curves of canopy photosynthetic use efficiency of light and n in responses to plant density in field-grown cotton. Field Crops Res. 203, 192–200. doi: 10.1016/j.fcr.2016.12.018

[B114] Yin M. Chen G. Luo H. Peng J. Gao X. Yuan C.. (2021). Effects of external IAA application on sucrose metabolism with cotton bolls and within-boll yield components. J. Nucl. Agric. Sci. 35, 1931–1940. (In Chinese)

[B115] Yin K. Gao C. Qiu J. (2017). Progress and prospects in plant genome editing. Nat. Plants 3, 17107. doi: 10.1038/nplants.2017.107 28758991

[B116] Yu H. Cao Y. Wang Z. Zhang J. Yang L. Zhao Z.. (2023). Identification of the most sensitive stage of cotton microspore development to water deficit and analysis of carbohydrate metabolism related to pollen viability. Environ. Exp. Bot. 206, 105168. doi: 10.1016/j.envexpbot.2022.105168

[B117] Zhang B. Geng W. Cui J. Mu K. Ma Y. Hu L. (2016a). Assessment of the biomass energy use potential of cotton byproducts in China. Cotton Sci. 28, 384–391. (In Chinese)

[B118] Zhang F. Jin X. Wang L. Li S. Wu S. Cheng C.. (2016b). A cotton annexin affects fiber elongation and secondary cell wall biosynthesis associated with Ca2+ influx, ROS homeostasis, and actin filament reorganization. Plant Physiol. 171, 1750–1770. doi: 10.1104/pp.16.00597 27255486 PMC4936584

[B119] Zhang C. Turgeon R. (2018). Mechanisms of phloem loading. Curr. Opin. Plant Biol. 43, 71–75. doi: 10.1016/j.pbi.2018.01.009 29448176

[B120] Zhang S. Wang H. Fan J. Zhang F. Cheng M. Yang L.. (2022). Quantifying source-sink relationships of drip-fertigated potato under various water and potassium supplies. Field Crops Res. 285, 108604. doi: 10.1016/j.fcr.2022.108604

[B121] Zhang D. Zhang Y. Li C. Dong H. (2019). On light and simplified cotton cultivation. Cotton Sci. 31, 163–168. (In Chinese)

[B122] Zhao L. Fang J. Xing J. Liu W. Peng P. Long H.. (2017). Identification and functional analysis of two cotton orthologs of MAX2 which control shoot lateral branching. Plant Mol. Biol. Rep. 35, 480–490. doi: 10.1007/s11105-017-1040-4

[B123] Zhao W. Wang R. Hu W. Zhou Z. (2019). Spatial difference of drought effect on photosynthesis of leaf subtending to cotton boll and its relationship with boll biomass. J. Agron. Crop Sci. 205, 263–273. doi: 10.1111/jac.12320

[B124] Zhao Z. Wang C. Yu X. Tian Y. Wang W. Zhang Y.. (2022). Auxin regulates source-sink carbohydrate partitioning and reproductive organ development in rice. Proc. Natl. Acad. Sci. U.S.A. 119, e2121671119. doi: 10.1073/pnas.2121671119 36037381 PMC9457257

[B125] Zhou Y. Zhang Z. T. Li M. Wei X. Z. Li X. J. Li B. Y.. (2015). Cotton (G ossypium hirsutum) 14-3-3 proteins participate in regulation of fibre initiation and elongation by modulating brassinosteroid signalling. Plant Biotechnol. J. 13, 269–280. doi: 10.1111/pbi.12275 25370928

[B126] Zhu X. G. Hasanuzzaman M. Jajoo A. Lawson T. Lin R. C. Liu C. M.. (2022). Improving photosynthesis through multidisciplinary efforts: The next frontier of photosynthesis research. Front. Plant Sci. 13. doi: 10.3389/fpls.2022.967203 PMC9563237 36247611

